# Contrasting Community Assembly Forces Drive Microbial Structural and Potential Functional Responses to Precipitation in an Incipient Soil System

**DOI:** 10.3389/fmicb.2021.754698

**Published:** 2021-11-23

**Authors:** Aditi Sengupta, Till H. M. Volkmann, Robert E. Danczak, James C. Stegen, Katerina Dontsova, Nate Abramson, Aaron S. Bugaj, Michael J. Volk, Katarena A. Matos, Antonio A. Meira-Neto, Albert Barberán, Julia W. Neilson, Raina M. Maier, Jon Chorover, Peter A. Troch, Laura K. Meredith

**Affiliations:** ^1^Department of Biology, California Lutheran University, Thousand Oaks, CA, United States; ^2^Biosphere 2, University of Arizona, Tucson, AZ, United States; ^3^Earth and Biological Sciences Directorate, Pacific Northwest National Laboratory, Richland, WA, United States; ^4^Department of Environmental Science, University of Arizona, Tucson, AZ, United States; ^5^Department of Geosciences, University of Arizona, Tucson, AZ, United States; ^6^Department of Chemical and Biomolecular Engineering, University of Illinois at Urbana-Champaign, Champaign, IL, United States; ^7^Department of Hydrology and Atmospheric Sciences, University of Arizona, Tucson, AZ, United States; ^8^School of Natural Resources and the Environment, University of Arizona, Tucson, AZ, United States

**Keywords:** metagenome, incipient soil, community assembly, landscape evolution, 16S amplicon sequencing

## Abstract

Microbial communities in incipient soil systems serve as the only biotic force shaping landscape evolution. However, the underlying ecological forces shaping microbial community structure and function are inadequately understood. We used amplicon sequencing to determine microbial taxonomic assembly and metagenome sequencing to evaluate microbial functional assembly in incipient basaltic soil subjected to precipitation. Community composition was stratified with soil depth in the pre-precipitation samples, with surficial communities maintaining their distinct structure and diversity after precipitation, while the deeper soil samples appeared to become more uniform. The structural community assembly remained deterministic in pre- and post-precipitation periods, with homogenous selection being dominant. Metagenome analysis revealed that carbon and nitrogen functional potential was assembled stochastically. Sub-populations putatively involved in the nitrogen cycle and carbon fixation experienced counteracting assembly pressures at the deepest depths, suggesting the communities may functionally assemble to respond to short-term environmental fluctuations and impact the landscape-scale response to perturbations. We propose that contrasting assembly forces impact microbial structure and potential function in an incipient landscape; *in situ* landscape characteristics (here homogenous parent material) drive community structure assembly, while short-term environmental fluctuations (here precipitation) shape environmental variations that are random in the soil depth profile and drive stochastic sub-population functional dynamics.

## Introduction

Microorganisms serve as the only biotic component of landscape evolution and co-evolve with hydrological dynamics to impact soil formation and development in incipient landscapes (Cockell et al., 2009; Nemergut et al., 2013; Bradley et al., 2014; Rime et al., 2016; Zaharescu et al., 2019). Microbial community responses to drying-wetting dynamics are variable and feed back into the co-evolving and interactive hydrobiogeochemical processes involved in landscape evolution. Some studies suggest that repeated drying and wetting cycles select for fast-growing microbes that use labile substrates released into the soil after rewetting (Jager and Bruins, 1975; Lund and Goksøyr, 1980; Scheu and Parkinson, 1994; Denef et al., 2001), and rapid drying and wetting events in particular may select for microbes adapted to rapid changes in water potential (Schimel et al., 1999) with osmoregulatory capacity (Parr et al., 1981; Kempf and Bremer, 1998). Drying and wetting episodes may increase soil microbial diversity, activity, and biomass (Schimel et al., 1999) by enhancing species coexistence through increased habitat connectivity, nutrient supply from microbial necromass, and hydration-controlled microbial motility within the soil environment (Fierer et al., 2003; Dechesne et al., 2010; Šťovíček et al., 2017). Contrasting results suggest decreases in microbial diversity upon rewetting due to increased competition with increased soil-pore connectivity (Engelhardt et al., 2018), selection for slow growing microbes under drying-wetting stress (Bottner, 1985; Van Gestel et al., 1993a,b), or present no significant change (Fierer et al., 2003).

Precipitation events leading to drying-wetting cycles differentially impact soil depths, with the surface generally experiencing greater dynamic conditions and faster drying-out rates than the deeper layers, which tend to be saturated for longer periods of time. Soil profiles are influenced by precipitation regimes including variable soil hydration properties (Šťovíček et al., 2017) and water availability in pores (Or et al., 2006) and biofilm formation in the soil microenvironment (Vos et al., 2013). Studies evaluating depth dependency of soil microbial communities in incipient soil systems in response to precipitation events are scarce (Sengupta et al., 2019), since most studies focus on near-surface interactions. However, generalized insights on soil microbial community composition with depth reveal decreasing diversity with depth (Fierer et al., 2003; Eilers et al., 2012; Hao et al., 2021) and variable community composition in the surface horizons, with relatively similar communities at deeper depths (Eilers et al., 2012; Tang et al., 2018).

Variable hydrologic regimes also affect microbial community assembly processes that govern spatiotemporal patterns in microbial community composition and establishment (Nemergut et al., 2013; Stegen et al., 2015; Graham et al., 2016b; Sengupta et al., 2019) and include deterministic (Graham et al., 2017a; shaped by variable or homogenous selection processes arising from biotic and abiotic conditions) and stochastic (Graham et al., 2017b; Zhou and Ning, 2017; shaped by dispersal or drift) effects. Dry-wet cycles have the potential to drive community assembly as powerful deterministic variables and/or homogenizing agents of dispersal (Evans and Wallenstein, 2012; Schimel, 2018; Zhang et al., 2019). The relative influence of stochastic and deterministic processes may lead to compositional differences in the communities (Stegen et al., 2015), which have been shown to indirectly affect the biogeochemical function (Nemergut et al., 2013; Graham et al., 2017b). Studies have shown that stochastic processes dominate in early successional soils, with progression toward deterministic assemblies in late succession (Ferrenberg et al., 2013; Dini-Andreote et al., 2015; Ortiz-Álvarez et al., 2018), while another study reported variable selection structuring incipient microbial community in incipient basaltic soil (Sengupta et al., 2019) suggesting inadequate understanding of assembly processes in incipient systems.

Assembly forces that drive compositional differences in taxa could also drive functional differences in those communities. This assumption has not been empirically tested to date and decoupling often observed between taxonomy and function (Burke et al., 2011; Louca et al., 2016a,b, 2018). Understanding functional assembly forces in landscapes is important, particularly in response to abiotic forcings such as dry-wet cycling and for predicting recovery of systems following major disturbances that “reset” the system (IPCC, 2014; Graham et al., 2021). Therefore, systematic studies at landscape scale are needed to understand how hydrological perturbations in the primary stages of landscape evolution impact the mechanisms of soil microbial community structure, function, and assembly.

We investigated impact of precipitation forcings on microbial community at the landscape scale in incipient basaltic soils by conducting a temporal experiment at the Landscape Evolution Observatory (LEO) facility housed at Biosphere 2 in University of Arizona (Pangle et al., 2015; Sengupta et al., 2017; Volkmann et al., 2018). The enclosed and controlled LEO environment houses three identical 330m^3^ artificial hillslopes filled with crushed basaltic tephra with well-defined physical boundary conditions including time-zero observations. To evaluate microbial community composition, diversity, and taxonomic assembly (using amplicon sequencing) and metabolic potential and functional assembly (using metagenome sequencing), we collected spatially distributed soil samples before and after a 48-day intense precipitation sequence. We hypothesized that after the precipitation sequence, community composition, richness, and diversity would increase as a result of increased water availability, and the carbon and nitrogen metabolic potential of the microbial community would be enhanced as indicated by the presence and abundance of CO_2_ and N fixation pathways. We also hypothesized that microbial community structural and potential functional assembly processes would shift from stochastic assembly pre-precipitation to deterministic assembly post-precipitation.

## Materials and Methods

### Landscape Evolution Observatory, Precipitation Regime, and Soil Coring

**Figure 1:**
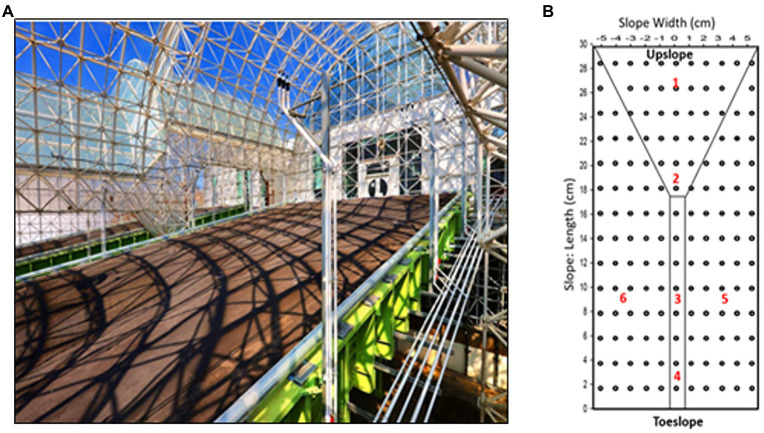
Landscape evolution observatory (LEO; **A**) upslope view of one of the hillslopes with **(B)** soil sampling locations (red numbers) and *in situ* moisture sensors (black).

Precipitation experiments were carried out on the three hillslopes (Figure 1; referred to as “East,” “Center,” and “West”) designed as replicates, with each hillslope (30m long, 11m wide, and 1m deep) positioned at a 10° slope, with a maximum slope of approximately 17° at the transition to the primary zone of convergence, and filled to a depth of 1m with crushed basaltic tephra (Pangle et al., 2015) of loamy sand texture. The hillslopes were established in 2012 and are sheltered from the outside environment, but are open to the internal environment of Biosphere 2. Time-zero characteristics of the basalt material included low organic carbon (7.03×10^−5^mg g^−1^) and nitrogen (4.33×10^−6^mg g^−1^), and the slopes were packed to a density of 1.59g cm^−3^ and average porosity of 39% (Pangle et al., 2015). LEO is fitted with a dense array of above- and below-ground sensors and samplers (Pangle et al., 2015; Sengupta et al., 2017) including 496 soil water content and temperature sensors (5TM, Decagon, Pullman, Washington, United States).

**Figure 2:**
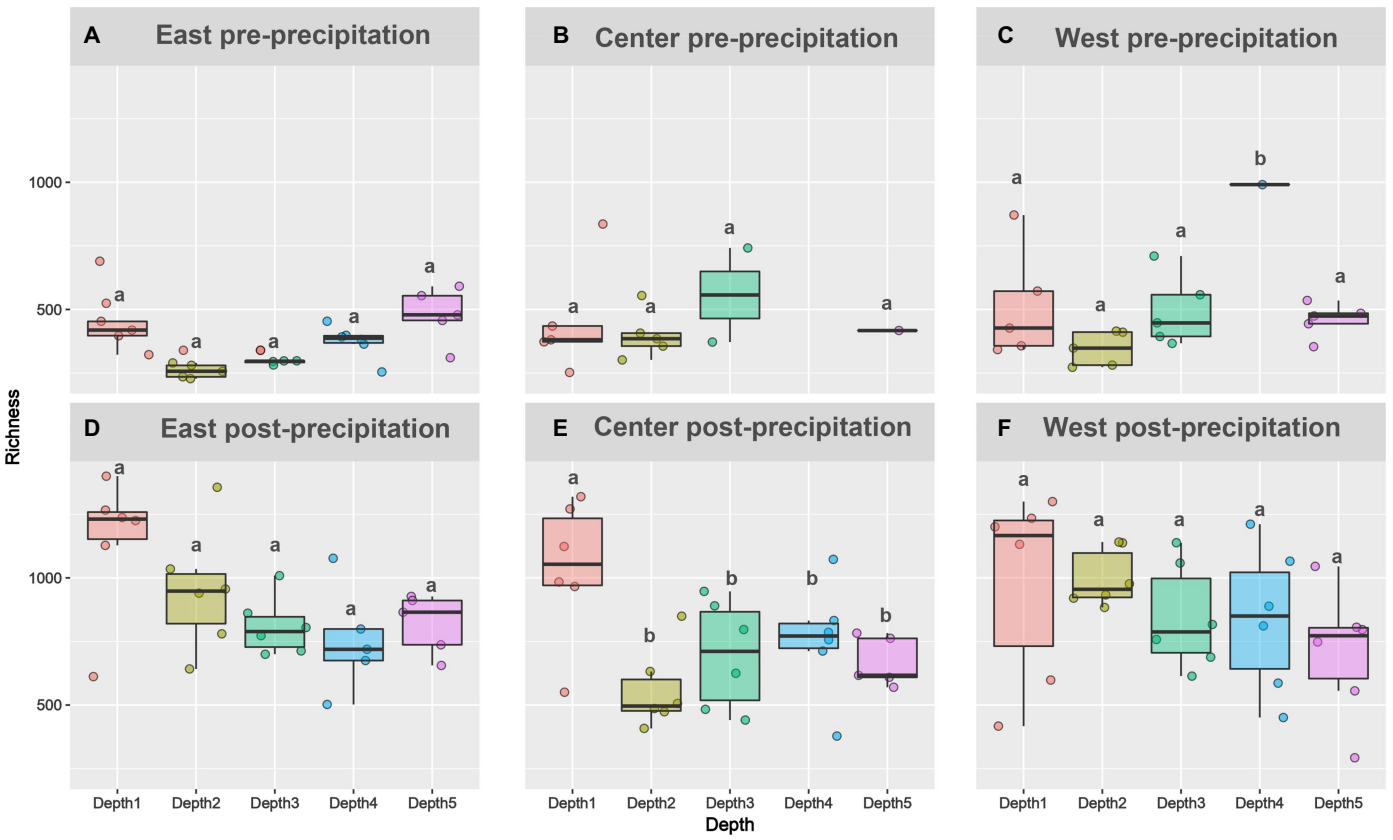
Alpha diversity increased following landscape wetting. Richness metric plotted for pre-precipitation **(A-C)** and post-precipitation **(D-F)** for each slope and depth. For depths with the same letter, the difference is not statistically significant within each slope per time point.

Following more than a year of no rain, the hillslopes were subjected to an intense period of precipitation using reverse-osmosis water during a PERiodic Tracer Hierarchy (PERTH) experiment from November 8, 2016, to December 26, 2016 (Graham et al., 2021). During this period, the precipitation cycle was repeated 15 times (Figure 2): A 3.5-daycycle consisted of two precipitation pulses, each of 3-h duration and 12mm/h rate and separated by a 7-h break. Small variations in the precipitation intensity over time or between landscapes were balanced by adjusting the duration of precipitation. The total precipitation per hillslope over the 1.5-month experimental period was ~1,200mm. Water table measurements were made using water pressure at different depths in the three hillslopes (Kim et al., 2020, 2021).

Soils were sampled by coring on November 6, 2016, pre-precipitation, and on December 21, 2016, to capture post-precipitation impacts. Six soil coring locations on each hillslope were chosen to represent topographic variability at LEO. The sampling locations were close to solution samplers and sensors (1.0–1.4m) to obtain complementary physicochemical measurements and modeled variables needed to conduct coupled hydrogeochemical analysis. Soil samples were collected from a personnel transport system above LEO that allows soil coring without stepping on the hillslope surface. Sampling location 1 (27m from the seepage face), 2 (19m from the seepage face), 3 (9m from the seepage face), and 4 (3m from the seepage face) represented upslope, convergence, mid-slope, and toe-slope regions, respectively, along the center of the slope, while locations 5 and 6 represented the side-slope regions 4m apart on each side of the mid-slope region (Site 3). To sample undisturbed soils, coring in December was offset by 0.5m to the right from November sites. A 1-m-long steel corer with 1-in internal diameter and fitted with 1×37–3/4-in plastic liners (AMS Inc., American Falls, ID, United States) powered by a drill was used to collect soil cores. The resulting hole in the soil was backfilled with an equivalent amount of original tephra material that had been aged in barrels by receiving precipitation water at similar rates as the hillslope soil. The plastic sleeve was extracted and sealed post-coring. In the lab, cores were sub-sampled at 12cm increments to retrieve 5 sub-depths [0–12cm (D1), 12–24cm (D2). 24–36cm (D3), 36–48cm (D4), and 48–60cm (D5)] totaling 180 samples (three hillslopes, six sites per hillslope, five depths per site, and two time points). Each sub-sampled core was homogenized and divided into halves for microbiology and geochemical analyses.

### Soil DNA Extraction and 16S rRNA Amplicon Gene Sequencing

Soil DNA was extracted using Fast DNA kit (MP Biomedicals®) following a modified protocol detailed in Sengupta et al. (2019). Paired-end sequencing (2×150bp) was performed on the bacterial and archaeal 16S rRNA gene using V4 (515F-GTGCCAGCMGCCGCGGTAA and 806R-GGACTACHVGGGTWTCTAAT primers) hypervariable region using the Illumina MiSeq platform (Illumina, CA, United States; Caporaso et al., 2012) for all samples and extraction blanks following Illumina sequencing library construction using the protocol previously published with modifications (Caporaso et al., 2012; Laubitz et al., 2016; Sengupta et al., 2019). Sequencing generated a total of 4,301,309 sequences for 180 samples after quality filtering raw reads. Data were analyzed by demultiplexing fastq-formatted sequences using split_libraries_fastq.py with a Phred quality cutoff of 20, followed by merging reads with a minimum of 25-base overlap (Caporaso et al., 2010). A summary of the sequences, post-merging and quality filtering was performed using mothur (v 1.25; Schloss et al., 2009). Samples with less than 5,000 quality-filtered sequences were dropped, resulting in a total of 158 samples for downstream analysis. Operational taxonomic unit (OTU) picking was done using UCLUST (Edgar, 2010), and sequence alignment was performed with PyNAST (Caporaso et al., 2010). Clustering was done with Greengenes database at 97% sequence similarity (DeSantis et al., 2006), chimera were removed with Chimera Slayer (Haas et al., 2011), taxonomy was assigned with RDP Classifier (Wang et al., 2007), and tree building was completed with FastTree (Price et al., 2010). OTUs with two or more sequences were retained. Unassigned OTUs as well as those identified as mitochondria and chloroplast were removed. All data files generated from QIIME workflow were imported into *R* environment program (v 3.4.0; R Core Team, 2014) for alpha and beta diversity estimation and visualization using *Phyloseq* (McMurdie and Holmes, 2013), statistical analyses using *vegan* (Oksanen, 2015), and differential abundance estimation using *DESeq2* (Love et al., 2014). Raw fastq files were deposited in NCBI’s SRP135809: PRJNA438505, per-sample raw sequence information, and per hillslope/time point observed OTU information is hosted on Figshare with accessibility information provided in the Data Availability statement.

### Environmental Parameters

Air-dried samples were analyzed for pH (U.S. EPA method 150.2), electrical conductivity (EC; U.S. EPA method 120.1), total carbon (TC), inorganic carbon (IC), organic carbon (OC), and total nitrogen (TN; U.S. EPA method 415.3; Sengupta et al., 2019). Soil temperature (°C) and soil water content (SWC; vol/vol) were measured using Decagon 5TM sensors with a dense sensor grid at 15-min resolution. The observations were interpolated onto the specific soil core locations (Figure 1B) and depths by 3D interpolation using piecewise polynomials. Soil moisture and temperature summary statistics were computed for each interpolated coring location at the instantaneous time of coring (e.g., SWC_mean_Instantaneous, Temperature_Instantaneous). Annual time points (e.g., SWC_mean_Annual, SWC_max_Annual, and Temp_Annual) were computed for the year prior to the respective coring date, for example, annual means for November included data from 11/08/2015 to 11/08/2016, while December means included data from 12/21/2015 to 12/21/2016. A dry duration variable (SWC_fractimedry_Annual) was calculated as the fraction of time soil water content was less than 5%.

### Data Analysis

Samples were assigned categorical variables including Time (pre- and post-precipitation), Slope (East, Central, West), Depth (D1, D2, D3, D4, and D5), and Location: Top (1), Convergence (2), MidCentral (3), Toe (4), MidRight (5), and MidLeft (6). Sequences were normalized across samples and richness assessed by performing pairwise ANOVA to evaluate significant differences in community composition for the whole community, individual time points (pre- and post-precipitation), and individual slopes. Beta diversity using the Bray-Curtis distance metric index was calculated. Nonparametric permutational multivariate ANOVA (PERMANOVA, 999 permutations, *vegan*) with strata/nestedness (slope and depth) was performed to evaluate significant effect of variables on the communities. Differential abundance of OTUs responding significantly to the precipitation treatment per-slope was detected using DESeq2, with significant (*α*=0.001) differential abundance evaluated as log2 folds change in the post- compared to the pre-precipitation samples.

### Community Assembly Processes

A previously developed null modeling framework was applied to estimate relative influences of different ecological processes (variable selection, homogenous selection, homogenizing dispersal, and dispersal limitation) on the community composition of samples (Stegen et al., 2012, 2013, 2015; Dini-Andreote et al., 2015). Community data rarefied to 9,207 sequences, and inferred phylogenetic relationships among OTUs were used to calculate between-community mean nearest taxon distance (βMNTD) metric (Stegen et al., 2013) for each pairwise community-to-community comparison within the pre- and post-precipitation samples. The βMNTD metric quantifies the phylogenetic distance between each OTU in one community and its closest relative in a second community. This metric is important for making ecological inferences based on phylogenetic turnover among closest relatives of microbes in a system with minor degree of organismal exchange (Stegen et al., 2013). Next, a null distribution of βMNTD was generated by randomizing phylogenetic relationships among OTUs and re-calculating pairwise βMNTD 999 times. This approach breaks association between OTUs and assumes no relationship (null model expectation). The degree to which βMNTD deviates from a null model expectation measures the relative influence of selection on community composition. For each pairwise comparison, homogeneous selection or variable selection was inferred as the ecological basis of community dissimilarity if the observed βMNTD value was significantly less or greater than the null distribution, respectively. The β-nearest taxon index (βNTI) was used to evaluate significance. βNTI expresses the difference between observed βMNTD and the mean of the null distribution in units of SDs with βNTI values<−2 or >+2 indicating significance. If *β*NTI is greater than 2, then variable selection occurs where two communities are more dissimilar than would be expected by random chance (e.g., when heterogenous environmental conditions between the compared communities result in different compositions; Graham et al., 2016a). Homogenizing selection is considered as the dominant process if *β*NTI is less than −2, which means the communities are more similar than could occur by random chance. For detailed understanding of the βMNTD and βNTI calculations, we refer readers to Stegen et al. (2013).

If observed βMNTD does not significantly deviate from the null expectation, then the observed compositional difference is not due to selection and may be due to either homogenizing dispersal or dispersal limitation. In these cases, a version of the Raup-Crick metric known as RC_bray_ (Stegen et al., 2013) is used. For each pairwise comparison, the null Bray-Curtis distribution is generated using 999 null model runs that simulated stochastic community assembly (see Stegen et al., 2013; for details). A value of RC_bray_<−0.95 indicates communities are more similar than expected, and when paired with |βNTI|<2, a dominant influence of homogenizing dispersal is inferred. Similarly, a value of RC_bray_>+0.95 indicates greater dissimilarity than expected, and when paired with a βNTI value that is non-significant (i.e., |βNTI|<2), a dominant influence of dispersal limitation is inferred. If, for a given pairwise comparison, neither null model is significant (i.e., |βNTI|<2 and |RC_bray_|<0.95), observed dissimilarity is not the result of any one process, and this situation is referred to as being “undominated” (Stegen et al., 2015). Mantel tests were performed to evaluate significant relationships (*p*≤0.05, *r*≥0.30). The R code for running the null models can be found here: https://github.com/stegen/Stegen_etal_ISME_2013.

### Metagenome Analysis

Based on the hydrologic history of the three hillslopes and storage-discharge relationships that showed East and West were more similar to each other than Center (Kim et al., 2020, 2021), representative samples from the East and West slopes were selected for metagenomic analysis to evaluate the impact of hydrological dynamics on microbial C and N cycling processes. A total of 24 samples were selected along three depth profiles: 0–12cm (D1), 12–24cm (D2), and 36–48cm (D4), two locations: the mid-convergence zone and the toe-slope near the seepage face, two time points (November and December), and two slopes (East and West). Metagenome sequencing was performed at the Department of Energy Joint Genome Institute (JGI) on an Illumina HiSeq 2,500 using Illumina Regular Fragment, 300bp library preparation method. Four samples failed to meet sequencing quality requirements and were dropped from further analysis. Sequences were obtained as per standard protocol outlined in Chen et al. (2021). Metagenomes are deposited in the JGI Integrated Microbial Genomes (60) under project ID 502880 and can be accessed through the Genomes OnLine Database (GOLD; Mukherjee et al., 2021). Information about the metagenome sequences is available as DataFile 2 on figshare.

Raw reads and assembled metagenomes were downloaded from IMG-JGI. Information about metagenomes, including gene counts and genome statistics, is provided in DataFile2. Assemblies were first filtered to retain contigs larger than 2,500bp. Assembled metagenomes were gene-called and translated using Prodigal (*prodigal -i [fasta] -a [output] -d [output] -p meta -m*; Hyatt et al., 2010) and then searched for the presence of marker genes corresponding to specific functional potential using HMMs obtained from PFAM and TIGRFAM (*hmmsearch --cpu 4 --noali -E 0.00001 --tblout [output] -o [output] [HMM] [amino acid fasta]*; Haft et al., 2001; Finn et al., 2016; Eddy, 2020). Metagenomes were mined for putative nitrate reducers identified using nitrate reductase subunit G (*narG*), putative nitrogen fixers identified using nitrogenase subunit H (*nifH*), and potential carbon fixers identified using RuBisCO large subunit (*rbcL*). The ribosomal protein subunit 3 (*rps3*) was also identified in each metagenome and used as a non-specific, general taxonomic marker gene that can be used to assess the broader microbial community. These genes were independently clustered at 100% identity using CD-Hit (*cd-hit-est -i [DNA fasta] -o [output] -c 1.00 -n 5 -M 8000 -d 0 -T 4*) to obtain unique sequence variants, which were subsequently used in phylogenetic tree generation. Each set of unique sequence variants was aligned using MUSCLE (Edgar, 2004) with default parameters and then trimmed using trimAl (with the *-gappyout* flag; Capella-Gutierrez et al., 2009). The trimmed alignments were analyzed using ModelTest-NG (Darriba et al., 2020; *-t ml* flag) to identify an optimal evolutionary model when generating the maximum-likelihood tree using RAxML-NG (Kozlov et al., 2019; *raxml-ng --all --msa [DNA fasta] --model GTR+I+G4 --bs-trees 100*). Additionally, reads were mapped to the unique sequence variants of each marker gene using bowtie2 (using the – fast setting) to obtain an estimate of abundance (Langmead and Salzberg, 2012).

The metagenomic data sets (e.g., approximate abundance and phylogenetic tree) were used to calculate βNTI for each marker gene following the approach described above yielding a total of four sets of null modeling results. Importantly, βNTI is quantitative; for example, while |βNTI|>2 indicates determinism, relating βNTI values to various metadata will allow us to examine the tendency for a community (or metacommunity) to be driven by specific environmental processes regardless of specific assembly processes. To evaluate the potential relationships between the assembly processes impacting these sub-populations, average βNTI values were calculated for each sample and correlated across null modeling data sets using ggpairs (GGally R package v. X; Schloerke et al., 2021). Two different sets of cross-null modeling correlations were performed: one where the βNTI averages were calculated across all samples, and another where βNTI averages were calculated while controlling for depth. Lastly, sub-population assembly was related to environmental parameters, while controlling for both depth and time. Here onwards, we refer to the 16S rRNA amplicon-derived βNTI results as taxonomic community assembly and metagenome-derived βNTI results as sub-community assembly.

## Results

### Soil Chemistry

Significant differences in soil chemistry were observed (Supplementary Figure 1), with mean and SDs provided in Supplementary Table 1. Post-precipitation, the landscape was more acidic than pre-precipitation conditions, with a soil pH drop of about 0.1–0.5 log units across slopes (East: W=236.5, *p*<0.05; Central: W=154.5, *p*<0.05; West: W=58, *p*<0.01). Electrical conductivity decreased sharply over the precipitation period, with all three slopes exhibiting 40–50% reduction (East: W=1, *p*<0.01; Central: W=22, *p*<0.01; West: W=20, *p*<0.01). Total carbon in the slopes decreased in the post-precipitation samples (East: W=1,025, *p*<0.01; Central: W=65, *p*<0.01; West: W=37, *p*<0.01) as well as organic carbon concentration decrease in the East slope (W=140, *p*<0.01), while inorganic carbon decreased in the Center (W=120, *p*<0.01) and West (W=72, *p*<0.01) slopes. Total nitrogen decreased post-precipitation (East: W=105, *p*<0.01, Central: W=57, *p*<0.01, West: W=92, *p*<0.01) in all slopes.

### Richness and Community Similarity Estimates

**Figure 3:**
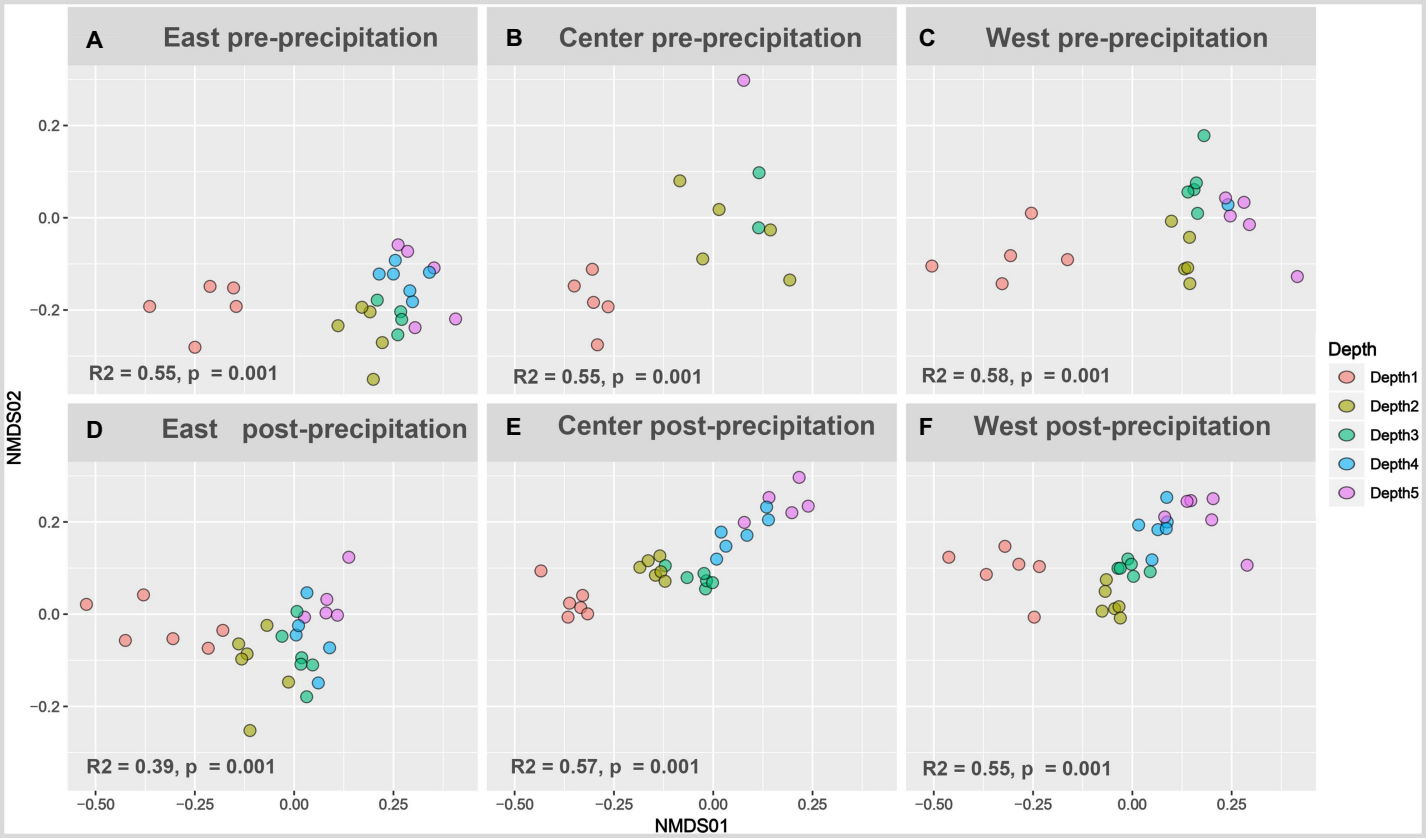
Beta diversity depth-dependent clustering increased with landscape wetting. Non-metric multidimensional scaling of pre-precipitation **(A-C)** and post-precipitation **(D-F)** samples plotted as Bray-Curtis matrix. Permutational multivariate ANOVA (PERMANOVA) performed on individual slopes nested for depth was significant for all time points (pre- and post-precipitation).

Amplicon sequencing generated 27 East pre-precipitation (1,084±613 OTUs), 18 Central pre-precipitation (1,282±1,248 OTUs), 23 West pre-precipitation (1,400±1,225 OTUs), and 30 each of East post-precipitation (3,202±1739 OTUs), Central post-precipitation (2,427±1,521 OTUs), and West post-precipitation (2,926±1,233 OTUs) samples. Phyla-level relative abundance changes were observed in all slopes, pre- and post-precipitation (Supplementary Figure 2). Richness increased significantly from after precipitation [East (*F*=38.16, *p*<0.005), Central (*F*=8.5, *p*=0.005), and West (*F*=17.48, *p*<0.005)] but did not vary significantly between depths during pre- or post-precipitation (Figure 3). Deviation from this trend was observed for the Central slope: samples had higher richness than samples collected at the other depths, post-precipitation (*F*=5.5, *p*=0.0026).

**Figure 4:**
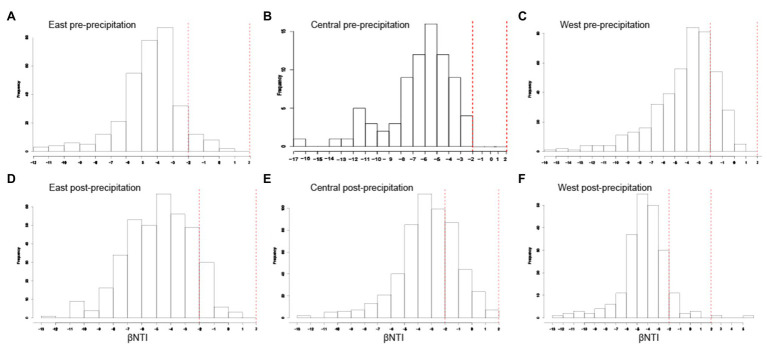
Homogeneous selection was the dominant structural community assembly process. Histograms of βNTI values for each hillslope pre- **(A-C)** and post-precipitation **(D-F)**. βNTI values<−2 indicate homogenous selection and those > +2 indicate variable selection.

Pre-precipitation samples showed little dissimilarity with depth – while surficial samples were distinct and separated from a diffused clustering of the deeper samples without strong depth-dependent stratification (Figure 4). The post-precipitation samples showed more gradual depth-dependent clustering, though surficial samples remained separate followed by distinct Depth 2 (12–24cm) and Depth 3 (24–36cm) clusters, while Depth 4 and 5 (36–60cm) were similar in their beta diversity. Length-dependent dissimilarity was observed only for the East slope post-precipitation (*R*^2^=0.25, *p*=0.007).

### Environmental Variables Impacting Community Dissimilarity

Permutational multivariate ANOVA results (Supplementary Table 2) showed that in pre-precipitation samples, TN, OC, EC, and SWC_annual significantly impacted community dissimilarity. Post-precipitation, TN, pH, EC, SWC_annual, and SWC_instantaneous, and Temperature_Instantaneous were significant. SWC_fractimedry impacted community composition at both time points and was the only variable that interacted with Depth to influence the community characteristics in pre-precipitation and the second one to influence post-precipitation characteristics (the second being instantaneous temperature).

### Differential Community Composition

**Figure 5:**
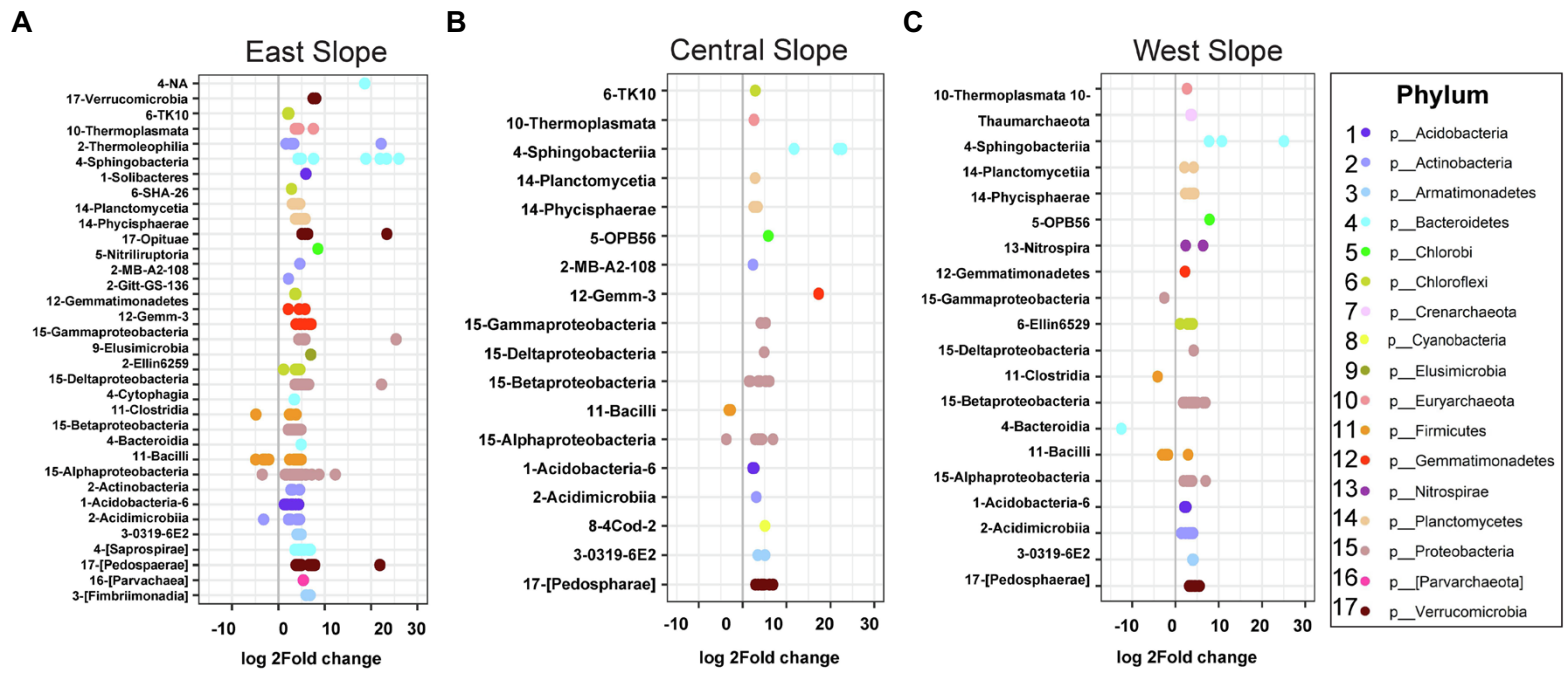
Microbial community membership shifted consistently with precipitation across slopes. Nested differentially abundant OTUs in **(A)** East, **(B)** Central, and **(C)** West hillslopes with differential enrichment of classes. In the figures, log2fold change greater than zero indicates enriched classes post-precipitation, while values less than zero indicate reductions relative to pre-precipitation.

Sequence analysis using DESeq2 revealed that microbial community membership shifted consistently with precipitation across slope (Figure 5). All three slopes showed similar trends of enriched OTUs, pre- and post-precipitation. *Firmicutes* (*c. Bacilli*) were enriched uniformly across all slopes pre-precipitation, while *Bacteroidetes*, *Gemmatimonadetes*, *Planctomycetes*, *Proteobacteria*, and *Verrucomicrobia* OTUs were enriched across all depths post-precipitation (Figures 5A–C). The East slope with a total of 231 differentially abundant OTUs categorized into 34 classes that were enriched in the post-precipitation samples, while four classes were enriched in pre-precipitation samples with OTUs predominantly belonged to phyla *Firmicutes* (class *Bacilli*) and *Actinobacteria* (class *Acidimicrobia*; Figure 5A). The Central slope had 54 differentially abundant OTUs with 18 differentially abundant classes of which class *Bacilli* and *Alphaproteobacteria* were enriched in the pre-precipitation samples (Figure 5B). The West slope included 81 differentially abundant OTUs grouped into 20 classes of which four (*Bacilli*, *Bacteroidia*, *Clostridia*, and *Gammaproteobacteria*) were enriched in the pre-precipitation samples (Figure 5C).

### Structural Community Assembly Processes

**Figure 6:**
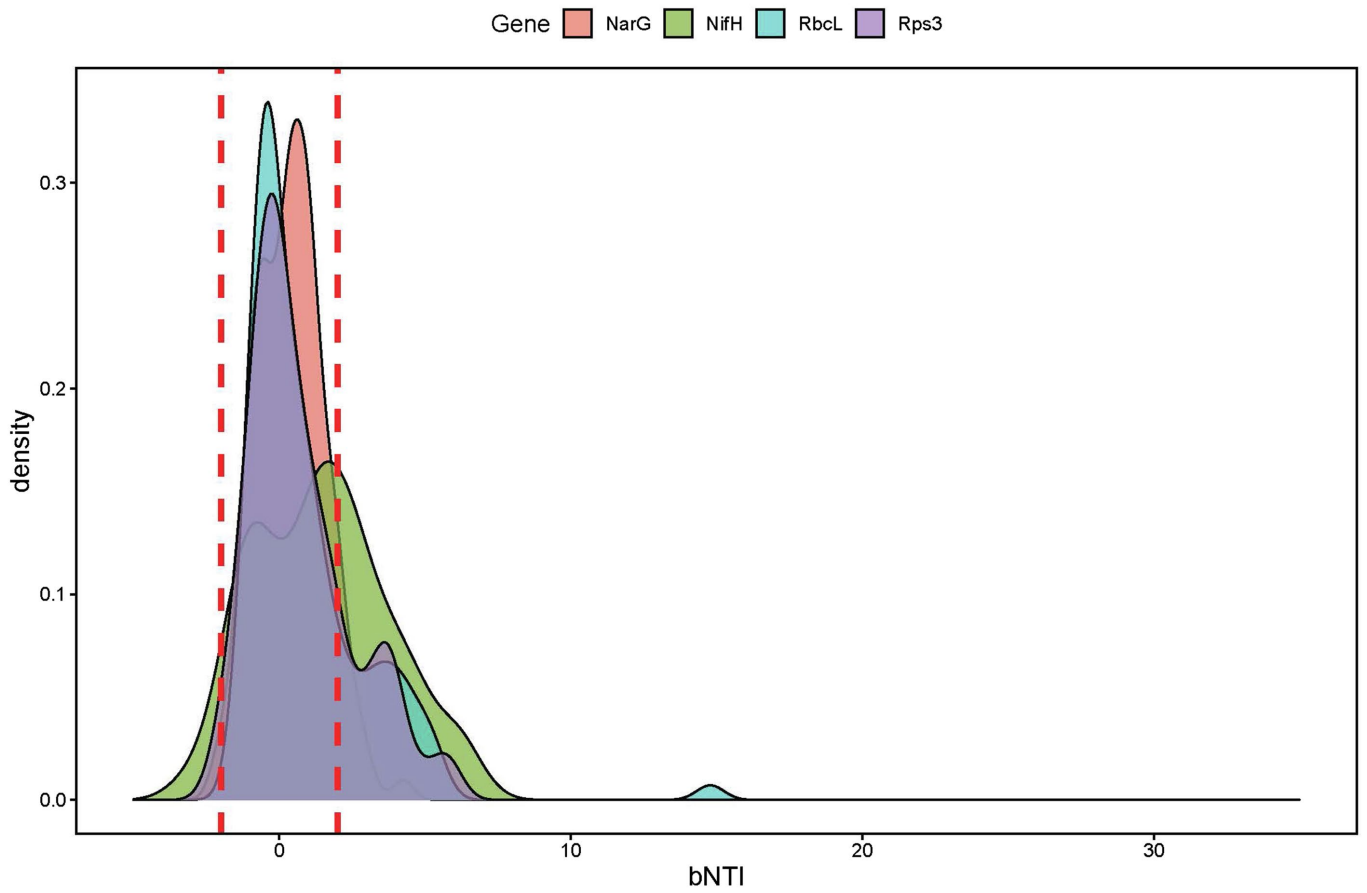
β-nearest taxon index values of the marker genes derived from metagenome sequences show stochastic processes governing the functional community assembly dynamics.

Pre- and post-precipitation communities exhibited deterministic influences of ecological assembly, with homogenous selection as the dominant structural community assembly process (βNTI<−2). Within each slope, community assembly appeared to be marginally driven by variable selection post-precipitation (as evidenced by the increase in frequency of βNTI toward +2 values) but largely remained homogenous (Figure 6). Mantel tests measuring the correlation between distance matrices of βNTI (dependent variable) and environmental variables (predictor variable) are provided in Supplementary Table 3. Strong positive correlation (*p*≤0.05, *r*≥0.30) was observed in the Central slope for DNA concentration, TC, TN, IC, SWC_mean_annual, and SWC_fractimedry_annual significant for pre-precipitation samples, while pH was significantly positively correlated in post-precipitation samples. Strong correlations were not observed for the East slope and only significantly positive pH and SWC_mean_annual in the West slope, post-precipitation. The RCBray results are not discussed since βNTI results were significant (dominantly <−2), indicating homogenous selection.

### Sub-Community Assembly Processes Defined by Functional Potential

**Figure 7:**
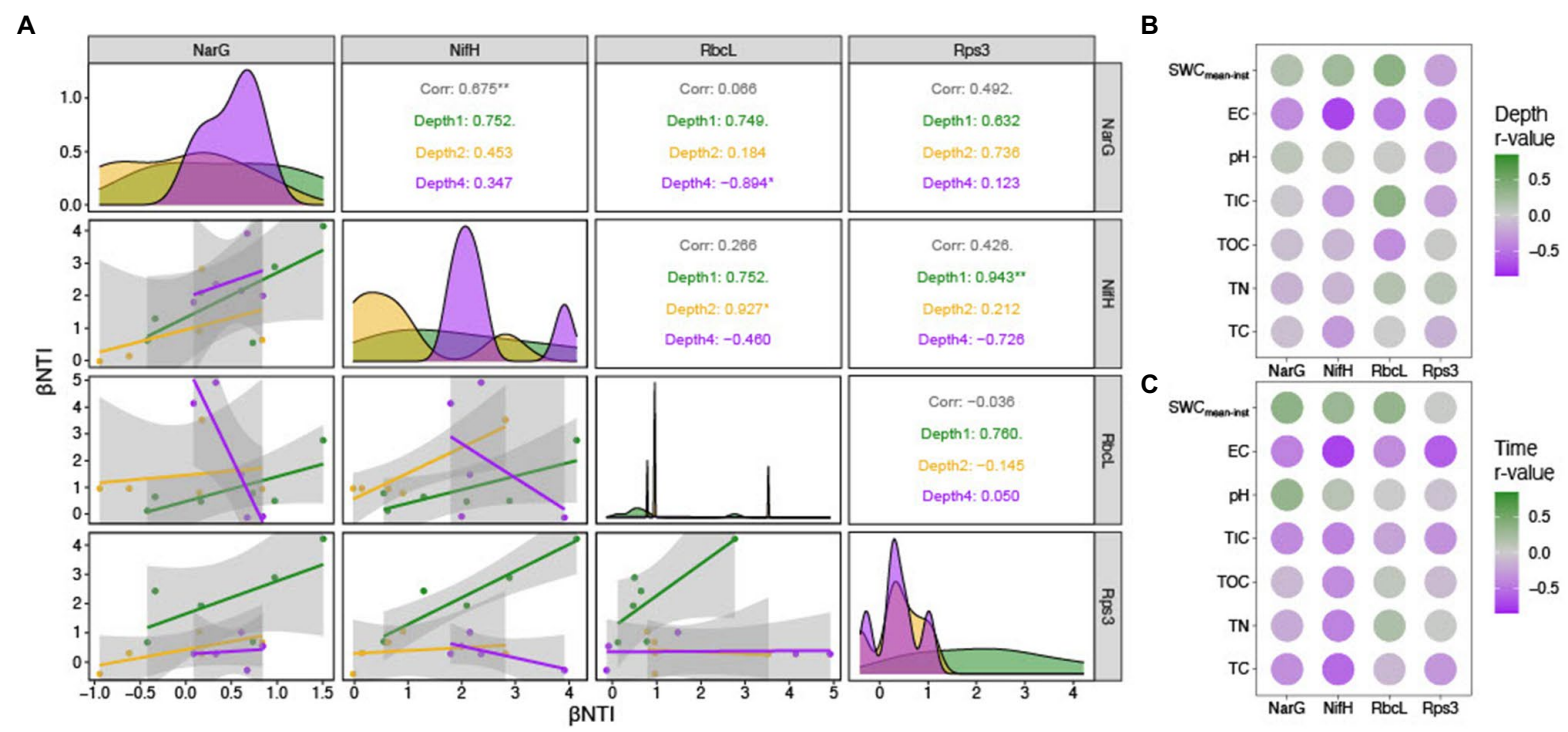
Stochastic processes governed functional community assembly, with related patterns between marker genes and with environmental variables. βNTI comparisons to investigate sub-population assembly dynamics. **(A)** Pairwise correlation plot between the average βNTI values for four sub-populations defined by nitrate reductase subunit G (narG), nitrogenase subunit H (nifH), RuBisCO large subunit (rbcL), and ribosomal protein subunit 3 (rps3) sequences. **(B)** Depth-controlled (i.e., only within-depth βNTI values were analyzed) correlation matrix with colors indicating the correlation coefficient. **(C)** Time-controlled (i.e., only βNTI values from the same date were analyzed) correlation matrix with colors indicating the correlation coefficient.

Ecological null modeling performed by calculating βNTI for the four marker genes derived from the metagenome sequences revealed that the majority of sub-community assembly dynamics appeared to be governed by stochastic processes (|βNTI|<2; Supplementary Figure 3). Given the quantitative nature of βNTI (e.g., higher absolute values trend toward determinism even if they are in the stochastic range), we performed pairwise correlations between the average βNTI values for each marker gene (Figure 7A) to further investigate the ecological assembly processes between these sub-populations. By relating the βNTI values for rps3 to βNTI values for the various functional marker genes, we can further evaluate whether sub-population assembly diverges from general community assembly. This is because rps3 is well-conserved that should be encoded by every microorganism within a given community (Hug et al., 2016). Given that depth-resolved differences were observed in beta diversity analyses by amplicon analysis, we calculated the mean for within-depth βNTI comparisons. For example, if a sample was from Depth1, only βNTI values between that sample and other Depth 1 samples were averaged. Upon correlation of all average within-depth βNTI values (*nar*G-*nif*H, *na*rG-*rbc*L, *nar*G-*rps*3, *nif*H-*rbc*L, *nif*H-*rps*3, and *rbc*L-*rps*3), we observed a significantly positive relationship for the *narG*-*nifH* comparison only (Figure 7A). Separating the values by depth and correlating again, however, revealed stark differences. We observed a significantly negative *narG*-*rbcL* relationship in Depth 4, a significantly positive *nifH*-*rbcL* relationship in Depth 2, and a significantly positive *nifH*-*rps3* relationship in Depth 1 (Figure 7A). While not significant, we also observed negative *nifH*-*rbcL* and *nifH*-rps3 relationships in Depth 4 (Figure 7A).

Potential environmental impacts on sub-population assembly were assessed by relating environmental variables to average within-depth and within-time βNTI values (Figures 7B,C). While many parameters were correlated in the within-depth comparisons, the *nifH*-TN, *rbcL*-TOC, and *rbcL*-TIC relationships were noteworthy (Figure 7B). The within-time correlations had a similar pattern of relationships, though the *nifH*-TN and *nifH*-TC comparisons were particularly strong (Figure 7C).

## Discussion

Soil microbes are the earliest biotic component on new landscapes and are impacted by the abiotic changes that occur over variable spatial and temporal scales (Phillips, 1988; Maurer and Gerke, 2016; Vitousek et al., 2016; Ma et al., 2017). For example, mineralogy of parent material remains essentially unchanged for years, while hydrologic process like soil water content undergo frequent perturbations and therefore impact microbial characteristics on shorter timescales. Our study evaluated changes in microbial community composition, diversity, and structural and functional assembly in an incipient landscape in response to a precipitation regime.

Microbial community composition and diversity changed with precipitation, with increasing alpha diversity and compositional similarity among communities. Depth-dependent structuring of the communities led to a more even community composition, similar to results reported in a meta-analysis of bacterial primary succession patterns (Ortiz-Álvarez et al., 2018). Community functional assembly through metagenomic null modeling revealed sub-populations experiencing depth-dependent assembly processes. For example, while we see a significant positive correlation between putative nitrate reducers and carbon fixers in Depth 2, this significance disappears and switches negative in Depth 4 (Figure 7A). This suggests that the sub-populations putatively involved in the nitrogen cycle and carbon fixation experience counteracting pressures at the deepest depths. Correlations with environmental parameters reveal that this divergence might be driven by TIC concentrations (Figure 7B). We observe that TIC is positively related to *rbcL*-based βNTI, but negatively related to *nifH*-based βNTI. As with the *nifH*-TN correlation, the relationship between TIC and *rbcL*-based βNTI might point to a direct selective interaction between RuBisCO diversity and available CO_2_, which is variable and often limiting in these weathering hillslopes (Cueva et al., 2019). We also observed that the *nifH*-TC and *nifH*-TN relationships were stronger when controlling for time than depth (Figures 7B,C). Given that precipitation was the differentiation factor through time, we suggest that this result was a potential signal for nutrient mobilization and that the time-controlled correlations better reflect the *nifH* sub-population dynamics. By having ready access to mobilized nutrients, the *nifH* sub-population better mirrored nitrogen behavior, either helping cause it or by responding to it.

We also observed coordinated assembly processes between the sub-populations of putative nitrate reducers and putative nitrogen fixers (Figure 7A). This pattern suggests that some common pressure (or set of pressures) is acting upon disparate ends of the nitrogen cycle (i.e., nitrate to nitrite and nitrogen gas to ammonia). By performing correlations with various environmental parameters, the amount of TN appears to be driving this coordination in part (Figure 7B). We hypothesize that total nitrogen content exerts a homogenizing effect on *narG*/*nifH* phylotypes due to differences in reaction efficiency (Nelson et al., 2020).

**Figure 8:**
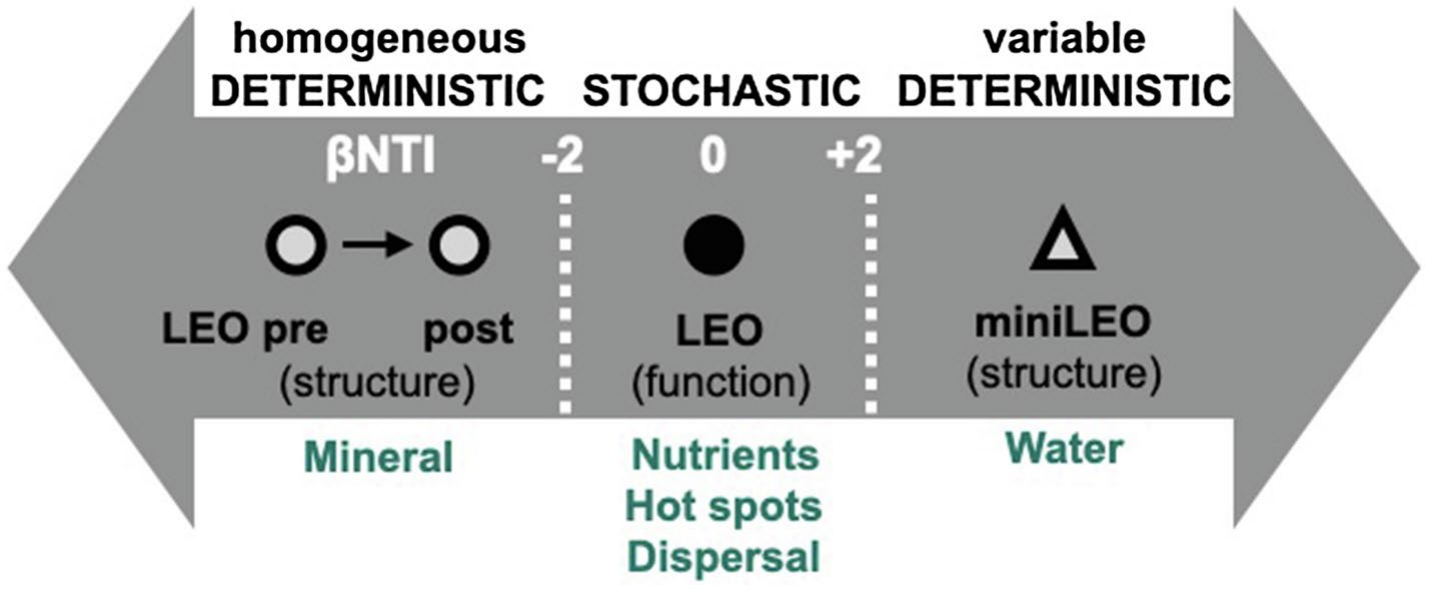
Conceptual depiction of microorganism assembly in an incipient system. Deterministic assembly processes select for structural composition, while stochastic processes driven by local environmental features shape functional assembly.

The assembly dynamics of sub-communities defined by functional potential was primarily responsible for community and sub-population according to metagenomic null modeling, homogenous selection (deterministic assembly with βNTI<−2) structured the community according to amplicon-based null modeling. This suggests no significant influence of variables measured in our system on community assembly processes. Hillslopes may have shifted from an initial stochastic community composition as indicated in primary succession of microbial communities (Ferrenberg et al., 2013) to a deterministic assembly influenced by homogenous environment selection. We posit that the mineralogy of the system is the most likely driver influencing the assembly metrics. Since mineralogy is not expected to shift within the 45day-span of sampling, it likely exerts a selection force that is homogenous between and within hillslopes. This contrasts with a study in the scaled-down (0.5m×2m×1.0m) version of LEO, called miniLEO that underwent prolonged 2-year intensive precipitation regime, where despite having similar depth-dependent community composition and structure to LEO, variable selection was the dominant assembly process (Sengupta et al., 2019). The strong homogenous selection in LEO suggests that in an incipient landscape, differential selection processes may occur at different landscape scales. It may also be likely that over a period of time, assembly processes are less susceptible to environmental variations at the landscape scale, while environment heterogeneity significantly influences microbial community assembly at smaller spatial scales (Figure 8).

In contrast, there appears to be no selection for functional potential at the landscape scale. Instead, local hotspots likely formed as a result of precipitation events and align function with local environmental features including nutrient, moisture, and temperature, giving rise to stochastic functional assembly. While mechanistic evaluation of sub-community assembly processes using metagenome data have not been documented so far or contrasted with structural community assembly features, there are a few potential explanations for this divergence between null modeling methods (e.g., fewer metagenomic samples, less diversity captured due to sequencing technique, *rps3* sequences approximate phylogeny differently than 16S rRNA sequences). An extension of this work may include evaluating the putatively active community (i.e., use rRNA and cDNA) once adequate biomass has been reached to determine community assembly response of the active members (Richter-Heitmann et al., 2020).

Precipitation events induced stratification of moisture conditions that likely influenced depth-dependent community restructuring in LEO. While not the focus of this research, parallel studies evaluating hydrological dynamics in the landscape revealed differing storage-discharge relationships in the slopes, suggesting differential water retention characteristics across depths (Supplementary Figure 4). Such heterogeneous hydrological structures that impact flow regime and accessibility of habitats can impact biological interactions (e.g., viruses and predation) and therefore shape the microbiome variability. Soil insulation against temperature fluctuations may also contribute to depth separation of the communities; post-precipitation community dissimilarity was influenced by instantaneous temperature (Supplementary Table 2).

The differential abundances of OTUs in our results showed a high abundance of *Firmicutes*, pre-precipitation, which are spore forming organisms and thrive in low-moisture conditions like the long pre-precipitation drying period (She et al., 2018). The increase in Proteobacteria abundance agrees with studies reporting similar increases following rewetting events (Castro et al., 2010). Microbial inputs to the incipient basaltic material are relatively low and include atmospheric deposition of microbes from the LEO space and from precipitation water. Cell counts in precipitation water (<10^3^ cells ml^−1^) were significantly lower than cell counts in water discharged from the slopes (>10^5.5^ cells ml^−1^; Honeker et al., in preparation). The increase in phyla post-precipitation (Figure 5) suggests that microorganisms present in the basaltic hillslopes are likely in a state of stasis with the potential to become active when in contact with water (Halverson et al., 2000; Niederberger et al., 2019), as has been observed in soils from colonization patterns of soil microbial communities following rewetting of dry soils (Crits-Christoph et al., 2013). However, we cannot differentiate whether precursor microbe proliferation in the lower depths or microbes flushed down during the rainfall events led to community restructuring.

Our study characterized microbial community composition and assembly in an incipient basaltic soil system subjected to precipitation in a controlled environment. Results showed that microbial community composition stratified in a depth-dependent manner, with communities exhibiting greater evenness after prolonged period of precipitation. We also hypothesize that mineralogy at the landscape scale likely impacted taxonomic community assembly, while sub-community assembly was influenced by local depth-dependent stratification of functional genes, likely due to depth-dependent moisture-driven dispersal events in the hillslopes. While differences between taxonomy and function have been reported where environmental conditions strongly influence functional group distribution but weakly influence taxonomic composition (Louca et al., 2016a), our results present a conceptual advance underlying this difference. Varying ecological processes may be an emergent feature in an incipient landscape and impact different components of the microbial community. We propose that contrasting assembly traits may shape different components of a microbial community in an incipient soil system and are largely driven by persistent (homogenous parent material) or fluctuating (periodic precipitation) processes in the system environment. The microbiomes of incipient systems may therefore assemble in a fundamentally different way than in established ecosystems, challenging the idea of a universal model for all successional stages of landscape evolution.

## Data Availability Statement

Amplicon-sequences are deposited in NCBI with accession number PRJNA438505. Metagenome sequences are publicly available in the Joint Genome Institute’s Integrated Metagenome/Genome database under project ID 502880. Soil environmental data, amplicon-sequence information, metagenome statistics, and βNTI functional ene correlations with time and depth are available in Figshare collection under embargo with https://doi.org/10.6084/m9.figshare.c.5516628.v1, and will be made public after publication. These files are also available from the corresponding author upon request.

## Author Contributions

AS, TV, and AM-N designed the experiment. AS conducted sample collection and processing, data analysis, and writing. RD and JS conducted the community assembly data analysis. KM and AM-N analyzed hydrology data. AB supported the amplicon-sequence analysis. KD supported soil sample collection and soil chemical analysis with MV. NA and AB helped in soil sample collection. JN, RM, JC, PT, and LM provided input on experimental design. RM, JC, PT, and LM secured funding. All authors contributed to the article and approved the submitted version.

## Funding

AS was supported by Biosphere 2 through the office of the Senior Vice President for Research Innovation and Impact at the University of Arizona. The authors gratefully acknowledge financial support from the Philecology Foundation and support of NSF-funded projects EAR-1344552, EAR-1340912, EAR-141709, and OIA-2121134. AS would like to acknowledge the intellectual inspiration provided by Ahana Sen in drafting the final version of the manuscript. AM-N would like to acknowledge the support received by the Brazilian Scientific Mobility Program promoted by CAPES. Additional funding support were provided by the Water, Environmental, and Energy Solutions (WEES) Initiative at the University of Arizona and by the Office of Research, Discovery and Innovation’s Accelerate for Success Grant at the University of Arizona. The authors would also like to acknowledge Daniel Laubitz at the University of Arizona Genomics Core for method development and sequencing of low-template samples. JS and RD were supported by the United States Department of Energy (DOE), Office of Biological and Environmental Research (BER), as part of Subsurface Biogeochemical Research Program’s Scientific Focus Area (SFA) at Pacific Northwest National Laboratory (PNNL). PNNL is operated for DOE by Battelle Memorial Institute under contract DE-AC06-76RLO 1830. Metagenome sequencing was conducted at the United States Department of Energy Joint Genome Institute, a DOE Office of Science User Facility, which is supported by the Office of Science of the United States Department of Energy under Contract No. DE-AC02-05CH11231 under Community Science Program Project 502880.

## Conflict of Interest

TV is currently employed by Accenture GmbH.

The remaining authors declare that the research was conducted in the absence of any commercial or financial relationships that could be construed as a potential conflict of interest.

## Publisher’s Note

All claims expressed in this article are solely those of the authors and do not necessarily represent those of their affiliated organizations, or those of the publisher, the editors and the reviewers. Any product that may be evaluated in this article, or claim that may be made by its manufacturer, is not guaranteed or endorsed by the publisher.
