# Supplementary material for: Contrasting Community Assembly Forces Drive Microbial Structural and Potential Functional Responses to Precipitation in an Incipient Soil System
Source: Front Microbiol. 2021 Nov 23;12:754698. doi: 10.3389/fmicb.2021.754698 (PMC8650109; doi:10.3389/fmicb.2021.754698)
Supplement: Supplementary file 1 [file Data_Sheet_1.PDF]

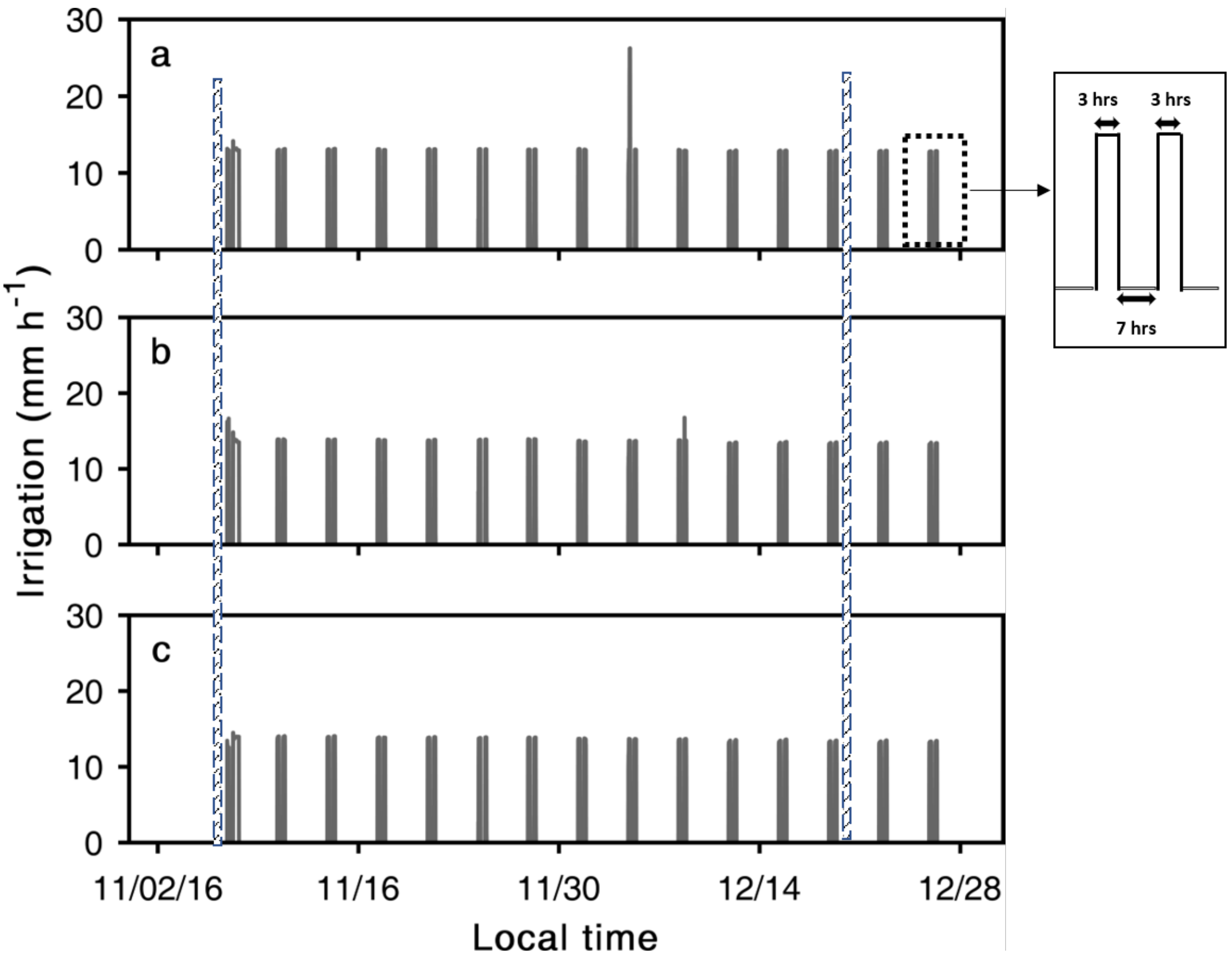

Supplementary Figure 1. Time series of measured precipitation rate supplied to the LEO East (a), Center (b), and West (c) landscapes over the duration of a periodic forcing experiment in November and December 2016. Dashed bars represent pre- and post-precipitation soil sampling times.

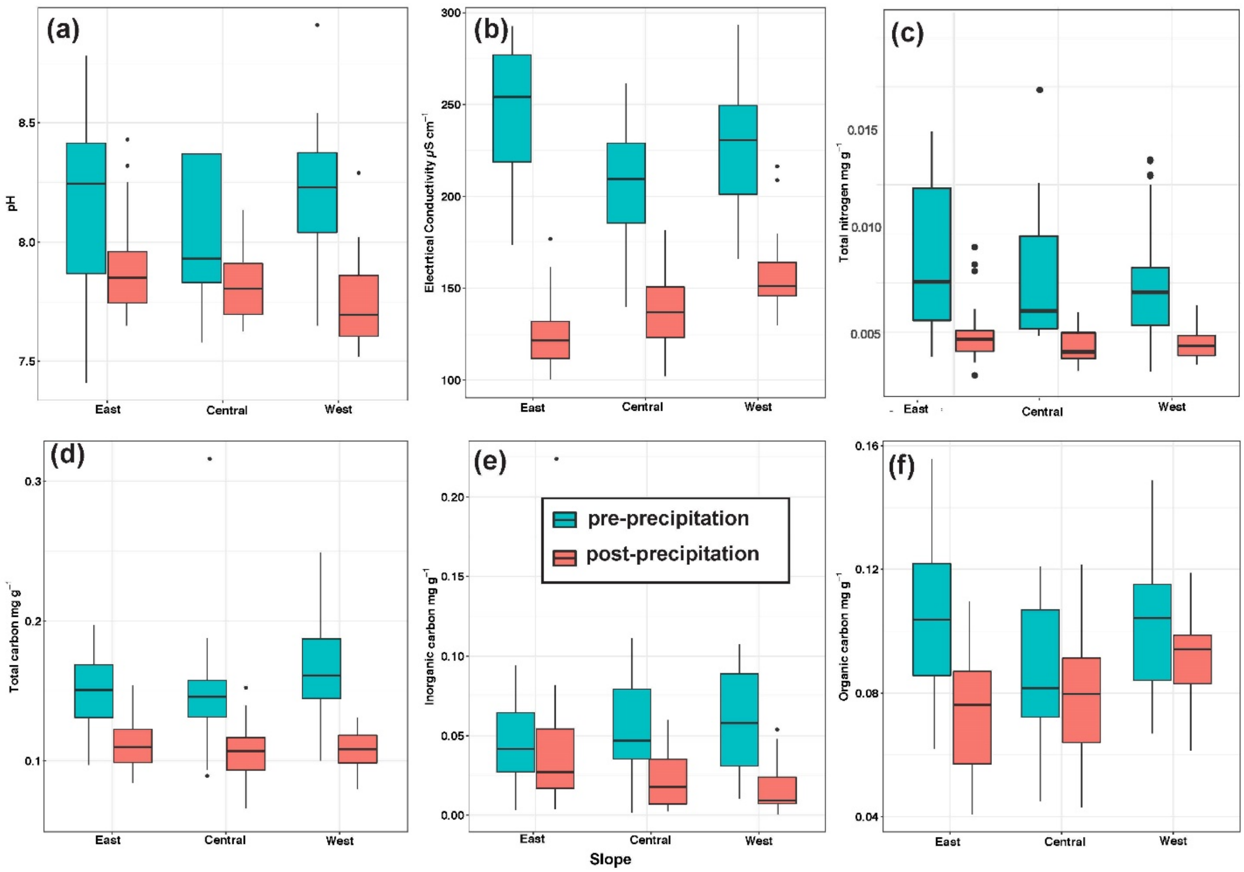

Supplementary Figure 2. Soil chemistry variables of samples in each slope, pre- and post-precipitation. Values have been averaged, with statistics reported in the results section.

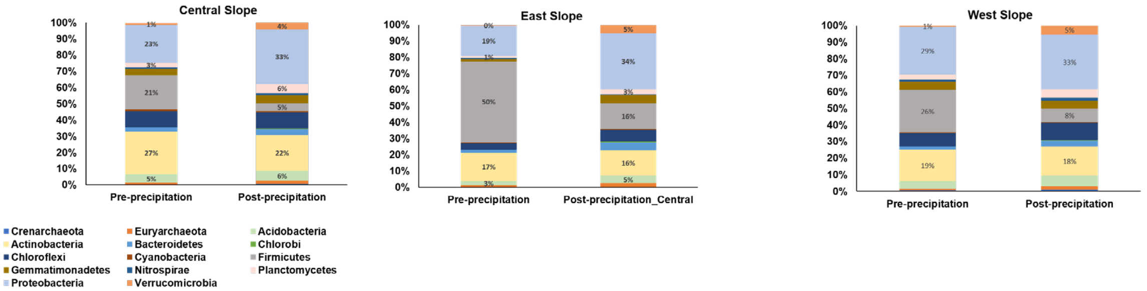

Supplementary Figure 3. Relative abundance of phyla-level OTU composition in the three hillslopes, pre- and post-precipitation.

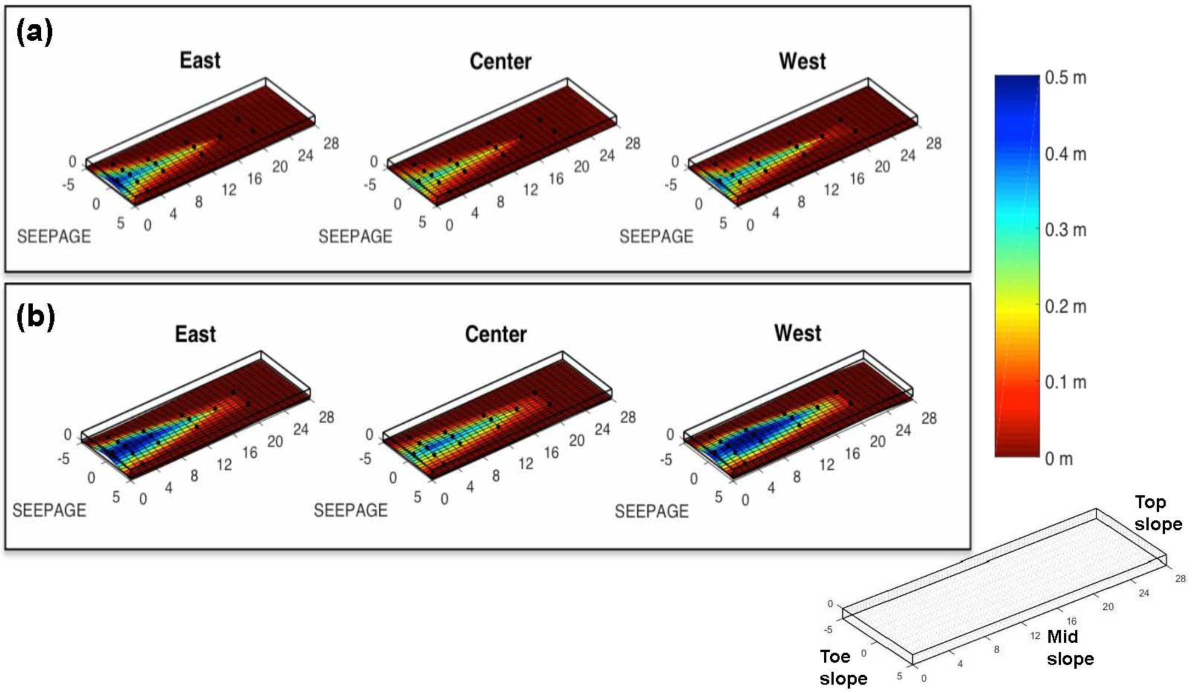

Supplementary Figure 4. Storage discharge relationship in the slopes show differential water retention characteristics across depths

Supplementary Table 1. Mean and standard deviation of soil chemistry da

Slope	Variable	Mean	Standard Deviation
East-pre	TC	0.148	0.028
	TN	0.008	0.004
	TOC	0.102	0.024
	TIC	0.037	0.025
	pH	8.12	0.391
	EC	243.126	36.173
East-pre	TC	0.109	0.017
	TN	0.004	0.002
	TOC	0.072	0.019
	TIC	0.026	0.023
	pH	7.899	0.193
	EC	124.24	18.848
Central-pre	TC	0.141	0.049
	TN	0.007	0.004
	TOC	0.083	0.023
	TIC	0.039	0.051
	pH	8.017	0.295
	EC	205.137	32.966
Central-post	TC	0.1	0.019
	TN	0.004	0.001
	TOC	0.077	0.019
	TIC	0.015	0.017
	pH	7.814	0.133
	EC	135.887	20.968
West-pre	TC	0.165	0.033
	TN	0.007	0.003
	TOC	0.101	0.021
	TIC	0.064	0.04
	pH	8.213	0.285
	EC	230.091	34.665
West-post	TC	0.108	0.013
	TN	0.004	0.001
	TOC	0.092	0.014
	TIC	0.016	0.015
	pH	7.744	0.178
	EC	157.591	19.122

Supplementary Table 2. PERMANOVA analysis of the effect of depth and environmental variables, their interactions on the distribution of OTUs in all slopes combined. * $p < 0.05$.

Pre-precipitation

Environmental variables	Df	SumOfSqs	R^2	F statistic	Pr(>F)
TC	1	0.4138	0.02219	1.4979	0.108
**TN**	1	0.698	0.03743	2.5662	**0.009**
**OC**	1	0.6576	0.03526	2.4123	**0.007**
IC	1	0.2199	0.01179	0.7875	0.698
pH	1	0.2527	0.01355	0.9066	0.528
**EC**	1	1.1473	0.06152	4.3266	**0.001**
SWC_mean_Instantaneous	1	0.3537	0.01897	1.276	0.202
**SWC_mean_Annual**	1	1.0858	0.05823	4.0806	**0.001**
**SWC_max_Annual**	1	1.9359	0.10381	7.6451	**0.001**
**SWC_fractimedry_Annual**	1	1.0916	0.05854	4.1038	**0.001**
Temp_Instantaneous	1	0.1218	0.00653	0.4339	0.998
Depth x TC	4	0.9408	0.05045	1.1323	0.253
Depth x TN	4	0.8585	0.04603	1.0276	0.422
Depth x OC	4	0.8897	0.04771	1.0824	0.299
Depth x IC	4	0.8528	0.04573	1.01	0.481
Depth x pH	4	0.9381	0.0503	1.1209	0.248
Depth x EC	4	0.9376	0.05028	1.1279	0.264
Depth x SWC_mean_Instantaneous	4	0.7612	0.04082	0.8924	0.683
Depth x SWC_mean_Annual	4	0.8041	0.04312	0.9796	0.502
Depth x SWC_max_Annual	4	0.7207	0.03865	0.8435	0.778
**Depth x SWC_fractimedry_Annual**	4	1.2687	0.06803	1.605	**0.009**
Depth x Temp_Instantaneous	4	1.0108	0.0542	1.2022	0.162

Post-precipitation

Environmental variables	Df	SumOfSqs	R^2	F statistic	Pr(>F)
TC	1	0.2733	0.0133	1.1864	0.251
**TN**	1	1.7356	0.08447	8.1193	**0.001**
OC	1	0.4055	0.01974	1.7718	0.032
IC	1	0.3413	0.01661	1.4865	0.083
**pH**	1	1.6928	0.08239	7.9015	**0.001**
**EC**	1	1.7766	0.08647	8.3297	**0.001**
**SWC_mean_Instantaneous**	1	0.791	0.0385	3.5235	**0.001**
**SWC_mean_Annual**	1	1.7606	0.08569	8.2476	**0.001**
SWC_max_Annual	1	0.3533	0.01719	1.5396	0.084
**SWC_fractimedry_Annual**	1	0.5071	0.02468	2.227	**0.012**
**Temp_Instantaneous**	1	1.0227	0.04978	4.6097	**0.001**
Depth x TC	4	0.6813	0.03316	0.99	0.483
Depth x TN	4	0.7095	0.03453	1.0299	0.406
Depth x OC	4	0.6539	0.03183	0.958	0.562
Depth x IC	4	0.7556	0.03678	1.1129	0.252
Depth x pH	4	0.739	0.03597	1.0954	0.278
Depth x EC	4	0.8066	0.03926	1.2293	0.12
Depth x SWC_mean_Instantaneous	4	0.6673	0.03248	0.9732	0.493
Depth x SWC_mean_Annual	4	0.8144	0.03964	1.2242	0.109
Depth x SWC_max_Annual	4	0.8293	0.04036	1.2168	0.136
**Depth x SWC_fractimedry_Annual**	4	1.7704	0.08617	2.7841	**0.001**
**Depth x Temp_Instantaneous**	4	1.0485	0.05103	1.5611	**0.007**

Supplementary Table 3. Correlation coefficient, r, of Mantel test between bNTI dissimilarity matrix and environmental variables for each slope and time point.

	**East, pre-precipitation**	**East, post-precipitation**	**Central, pre-precipitation**	**Central, post-precipitation**	**West, pre-precipitation**	**West, post-precipitation**
**Environmental Variable**	**Correlation coefficient, r**					
DNA concentration	0.08	0.23	**0.54**	0.17	0.11	0.08
TC	0.21	0.02	**0.74**	0.06	0.08	0
TN	0.02	0.23	**0.60**	0.18	0.01	0.25
OC	0.17	0.14	0.08	0.04	0.09	0.13
IC	0.05	0.03	**0.70**	0.11	0.04	0.06
pH	0.04	0.02	0.18	**0.32**	0.1	**0.32**
EC	0	0.16	0.05	0.08	0.1	0.24
SWC_mean_instantaneous	0.1	0.04	0.26	0.02	0.16	0.16
SWC_mean_annual	0.24	0.08	**0.39**	0.22	0.21	**0.38**
SWC_max_annual	0.05	0.04	0.12	0.02	0.26	0.12
SWC_fractimedry_anual	0.08	0.18	**0.42**	0.03	0.12	0.17
Temperature_Instantaneous	0.1	0.16	0.03	0.04	0.02	0.14
Temperature_Annual	0.18	0.17	0.03	0.05	0.15	0.13
